# Classical and Neural Network Machine Learning to Determine the Risk of Marijuana Use

**DOI:** 10.3390/ijerph18147466

**Published:** 2021-07-13

**Authors:** Laura Zoboroski, Torrey Wagner, Brent Langhals

**Affiliations:** 1 Data Analytics Certificate Program, Graduate School of Engineering and Management, Air Force Institute of Technology, Wright-Patterson AFB, Dayton, OH 45433, USA; laura.r.zoboroski.civ@mail.mil (L.Z.); brent.langhals@afit.edu (B.L.); 2 The Perduco Group (a LinQuest Company), Dayton, OH 45433, USA

**Keywords:** cannabis, marijuana, neural network, personality traits, prevention, THC

## Abstract

Marijuana is the most commonly abused drug for military personnel tested at the Air Force Drug Testing Laboratory. A publicly available dataset of drug use, personality trait scores and demographic data was modeled with logistic regression, decision tree and neural network models to determine the extent to which marijuana use can be predicted using personality traits. While the logistic regression model had lower performance than the neural network model, it matched the sensitivity of prior work (0.80), achieved a high level of significance (*p* < 0.05) and yielded valuable inferences. It implied that younger, less educated individuals who exhibit sensation-seeking behavior and are open to experience tend to be at higher risk for THC use. A method for performing an iterative multidimensional neural network hyperparameter search is presented, and two iterations of a 6-dimensional search were performed. Metrics were used to select a family of 8 promising models from a cohort of 4600 models, and the best NN model’s 0.87 sensitivity improved upon the literature. The model met an f1 overfitting threshold on the test and holdout datasets, and an accuracy sensitivity analysis on a holdout-equivalent dataset yielded a 95% CI of 0.86 ± 0.04. These results have the potential to increase the efficacy of drug prevention and intervention programs.

## 1. Introduction

All military services within the Department of Defense adhere to regulations on the use and misuse of drugs. The United States Air Force (USAF), with many critical missions, is particularly strict concerning such regulations. The Air Force Drug Testing Laboratory (AFDTL) currently tests Air Force and some Army military personnel for 22 different drugs or drug metabolites. Historically, the highest number of positive results are for marijuana, also known as cannabis, or by its psychoactive component, tetrahydrocannabinol (THC). When considering all positive drug tests between March and June 2020, the percentage of THC-positive results was 64%, while the next highest percentage of positive results was 18% for amphetamines. Although the amount of THC-positive results represents only 0.84% of the total population tested during this four-month time frame, there were still 1022 positive results [1]. While not static, these numbers represent typical proportions.

The prevalence of THC use in the military corresponds to the prevalence of THC use in the United States. In an annual survey conducted by the University of Michigan’s Institute for Social Research, marijuana has consistently been the most commonly used illicit drug [2]. This trend has led researchers to study the extent to which certain personality traits can identify risk factors for THC use in individuals [3,4,5,6,7,8]. Subsequently, intervention methods focusing on identified risk-prone personality traits to reduce the prevalence of THC use, particularly among adolescents, have also been studied [9,10].

Accurate prediction of drug use supports the USAF Drug Demand Reduction Program, the primary goal of which is to ensure mission readiness, as drug abuse “… seriously impairs accomplishing the military mission and is a threat to the health, safety, security and welfare of the total force [11].” The Air Force Alcohol and Drug Abuse Prevention and Treatment (ADAPT) Program [12] and the Army Substance Abuse Program (ASAP) [13] include drug prevention as an integral duty within the scope of their missions, as directed by the Department of Defense [14]. The results of this study could assist both the ADAPT Program and the ASAP in their prevention goals by providing an innovative selective prevention process to discover individuals at greater risk for substance use disorders [15]. Although a continuing topic of research and debate, Williams argues that THC use may also increase the risk for more serious opioid use disorder [16]. With exponentially increasing fatalities from opioid overdoses over the past four decades [17], targeted prevention methods enabled by the modeling in this work may help to reduce lethal overdoses in addition to mitigating military career impact. 

An additional goal of this study is to determine whether a neural network (NN) model can improve upon existing logistic regression (LR), or decision tree (DT) algorithms to predict THC use. Neural networks have been successful, and often superior to other algorithms, when making predictions in situations such as the success rates in a smoking cessation program [18], incidence of metabolic syndrome [19] and infectious disease surveillance [20]. Although it is becoming more common to use machine learning techniques to predict the risk of drug use or abuse [3,4,5,6,7,8,21,22,23], few researchers have employed NN models [21,23]. In a literature search, while four studies specifically modeled THC prediction using machine learning techniques, none employed a NN model. Three of these studies utilized a form of LR [5,6,7] and one used a DT [8], with moderate to high predictive accuracy. These studies incorporated personality traits along with additional risk factors. 

Haug et al. explored the initiation of THC and other drug use in young adult males using hierarchical logistic stepwise regressions, reporting a Nagelkerke’s R^2^ value of 0.11. Of the personality traits included, sensation-seeking behavior was revealed as a positive associated risk factor for the initiation of cannabis use [7]. Spechler, in his thesis employing multimodal data to predict THC use in adolescents, obtained mean area under the curve (AUC) measures of 0.71 for males and 0.81 for females utilizing LR with elastic-net regularization. This study included 2413 predictor features, and of those measuring personality, increased novelty seeking and decreased conscientiousness were found to contribute to early THC use [6]. Rajapaksha et al. utilized LASSO LR to predict cannabis use disorder (CUD) in known, regular THC users, achieving an overall accuracy of 0.66 and AUC of 0.65. Considering personality predictors, they found that CUD risk was higher in younger individuals prone to sensation seeking, having higher openness to experience and lower conscientiousness scores [5]. Fehrman et al. found that a DT model produced the best predictive results for the risk of 18 different drugs in their study, reporting a sensitivity of 79% and specificity of 80% for the risk of THC use [8]. Focusing primarily on personality traits, they report higher neuroticism, openness, impulsivity and lower conscientiousness and agreeableness scores to suggest a higher risk for THC use.

## 2. Background

This study uses the Fehrmen et al. dataset to determine the relationship of various personality traits to drug use [24]. Questions on the use of 18 different legal and illegal drugs, along with questions to determine personality traits from the Revised NEO-Five Factor Inventory (NEO-FFI-R), Barratt Impulsiveness Scale (BIS-11) and Impulsiveness Sensation-Seeking Scale (ImpSS) were employed. The NEO-FFI-R consists of 60 questions which assess the personality traits of neuroticism, extraversion, openness to experience, agreeableness and conscientiousness. The BIS-11 asks 30 questions gauging impulsiveness, and the ImpSS contains 19 questions measuring both impulsiveness and sensation-seeking behavior [8]. The results of this anonymous online survey were compiled into a dataset publicly available from the University of California, Irvine (UCI) Machine Learning Repository [24]. Although this dataset includes information on the use of various drugs, the present work focuses only on THC use.

**Figure 1:**
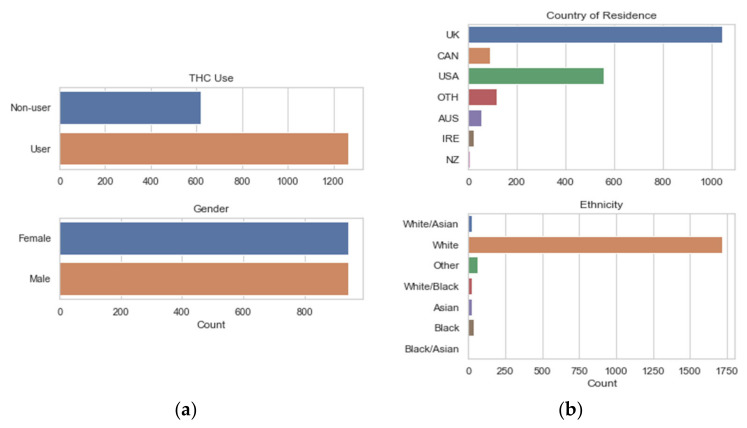
Distribution of the *THC Use* response variable (**a**) and other nominal categorical variables (**a**,**b**). UK: United Kingdom, CAN: Canada, USA: United States, OTH: Other, AUS: Australia, IRE: Ireland, and NZ: New Zealand.

The dataset contains 1885 observations, with responses from six labeled countries plus others combined; primarily the United Kingdom (UK) and the United States (USA). THC use is divided into seven classes based on the time frame of use, and these data were transformed into a binary categorical variable: non-users and users. THC non-users (0) have either never used THC or have not used in the last 10 years, with all others categorized as users (1). Gender was also represented by a binary categorical variable of female (0) and male (1). The distributions of THC users and the categorical variables used in this study are shown in Figure 1.

**Figure 2:**
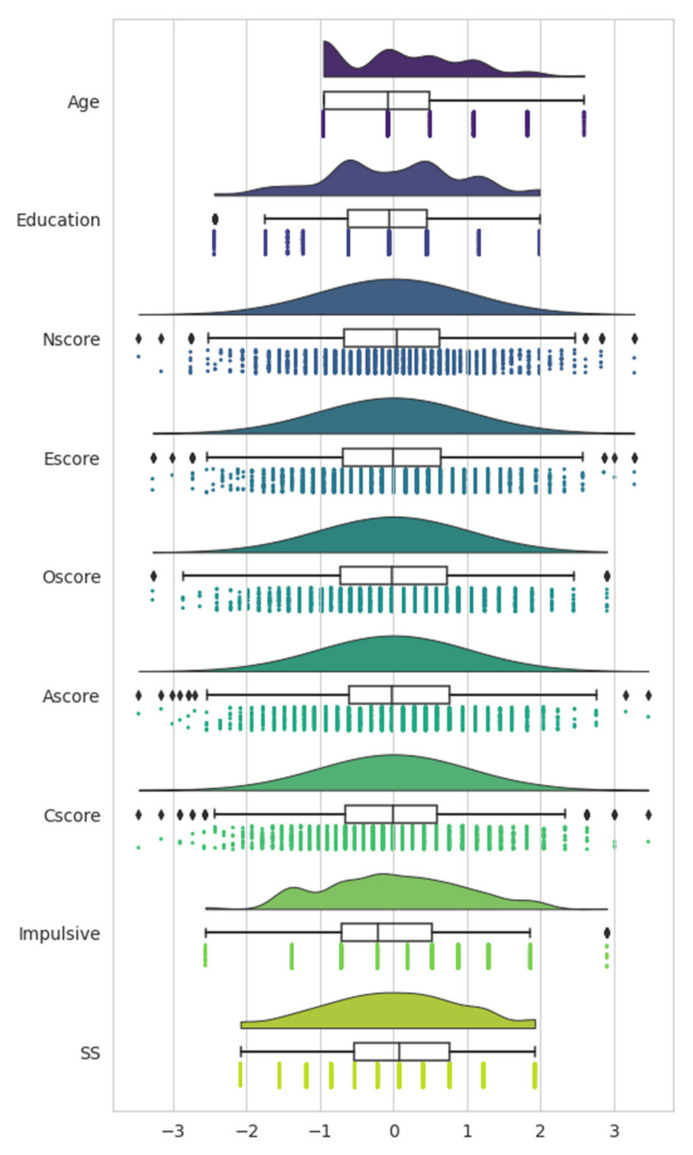
Distribution of quantified ordinal variables.

Data understanding for the remaining features is gained from the Figure 2 raincloud plot, which combines a box plot with a probability distribution function for each feature [25]. Figure 2 data are normalized; the age is categorized by 6 age groups ranging from 18 to over 65, and the education level ranges from those who left school prior to turning 16 to those with a doctorate degree. The distribution of the personality trait variables is quasi-normal: Nscore measures neuroticism; Escore, extraversion; Oscore, openness to experience; Ascore, agreeableness; Cscore, conscientiousness; Impulsive, impulsivity; and SS measures sensation-seeking behavior [24]. Numerous outliers are visible in Figure 2, as shown by the diamonds that are outside the extended interquartile range markers. These datapoints were retained to maximize the ability of the model to generalize upon unseen data.

Participant age starts at 18, but as shown in the top subplot of Figure 2, this dataset includes more older individuals than are typically included in drug use studies. The education level of participants and personality trait variables are all near-normally distributed. The ratio of THC users to non-users is approximately 2:1. Among the demographic data collected, male and female responses are nearly equal, but the data on country of residence and ethnicity are particularly skewed, as seen in Figure 1. Most responses came from the UK and USA, and the overwhelming ethnicity represented is White.

## 3. Method

The primary method used in this work is the cross-industry standard process for data mining (CRISP-DM), with the phases of data understanding, data preparation, modeling and evaluation [26]. Data preparation, LR/DT/NN modeling and model evaluation were conducted in Python using a GPU-enabled Google Colaboratory environment, and the Scikit-learn, Keras and TensorFlow frameworks. The data understanding phase was described in the preceding Background section of this paper.

### 3.1. Data Preparation

Fehrman et al. quantified their dataset using categorical principal component analysis on the nominal variables (country, ethnicity), and polychoric correlation on the remaining ordinal variables [8]. This created a fully quantified (non-categorical) dataset apart from the response variable.

The values for response variable THC use were changed from seven classes based on frequency of use to two classes, “user” and “non-user.” Non-users include those who have never used THC or used THC over a decade ago, and are labeled as “0”. Users include those who have used THC within the last decade to within the last day, and are labeled as “1.”

The LR and DT models used the quantified and standardized features shown in Figure 1 and Figure 2, except for ethnicity because of its unbalanced distribution. Due to the increased capacity of the NN model, a one-hot encoded transformation of the country and ethnicity features was included.

### 3.2. Metrics

In the dataset, the positive class is the THC user. While accuracy on both classes is desirable, for the purposes of this study it is considered more important to avoid false negatives (FN), where a user is miscategorized as a non-user. In this case, a person at risk for THC does not receive information or support. A corollary to this statement is that a high true positive rate is important. There is negligible consequence for a false positive (FP), where a non-user is misclassified as a user; here, the only drawback is that the member may receive information on avoiding drug use when it is not needed. 

Due to the consequences of false negatives and the importance of true positives, the main evaluation metrics used to compare models are the FN count and sensitivity, which is the ratio of true positives over the sum of true negatives and false positives. Sensitivity, also known as recall, allows comparison to prior work.

The f1 metric, defined as the harmonic mean of recall and precision, was selected to compare models due to the unbalanced nature of the dataset where 67% of the labels are positive. For a complete comparison, accuracy and AUC are also presented for all models. 

### 3.3. Logistic Regression Model

The logit algorithm within the Python Statsmodels framework, and the LogisticRegression algorithm with the SciKit-learn (sklearn) framework were used to model the dataset. Performance metrics were calculated using methods within the sklearn Metrics framework. The data were divided using a 70/30 train–test split to evaluate and compare the predictive ability of each model and check for overfitting of the model. Adding a constant value for the intercept increased the model’s accuracy. However, no L1, L2 or elasticnet regularization was added. The default values of quasi-Newton lbfgs solver, 100 maximum iterations and no class weights were used.

The log likelihood ratio *p*-value is <0.05 level of significance, indicating that the model is suitable for predicting the response variable. Although four variables—Gender, Nscore, Ascore and Impulsive—yielded *p*-values above a 0.05 level of significance, they were retained for more relevant comparison to the NN model. Accuracy and AUC scores of the train and test sets were monitored to assess overfitting.

### 3.4. Decision Tree Model

For the DT model, the data were modeled with the DecisionTreeClassifier algorithm within the sklearn Tree framework, and the model was evaluated using the same sklearn methods as the LR model. The classification DT model was modeled using the Gini index attribute selection measure and the same features as the LR model. A 70/30 train–test split was used to monitor overfitting, and a sweep of leaf nodes was conducted to determine the optimal model. The default value of no class weights was used, without limitations on the number of samples per split or the number of samples per node.

### 3.5. Neural Network Model

NN models were created using the Keras framework and sequential architectures [27]. The modeling effort was monitored for overfitting using a 70%/15%/15% train/test/holdout split, and a checkpointing algorithm was used during training to save the model each time the loss metric improved on the validation dataset. The stochastic gradient descent (SGD) optimizer algorithm was evaluated and compared to the Adam optimizer, which has been shown to be effective for both shallow and deep neural networks. The ReLu activation function was utilized for the input layer and the output layer utilized the sigmoid activation function. Binary cross-entropy was chosen as the loss function, and the f1 metric was monitored for overfitting.

**Figure 3:**
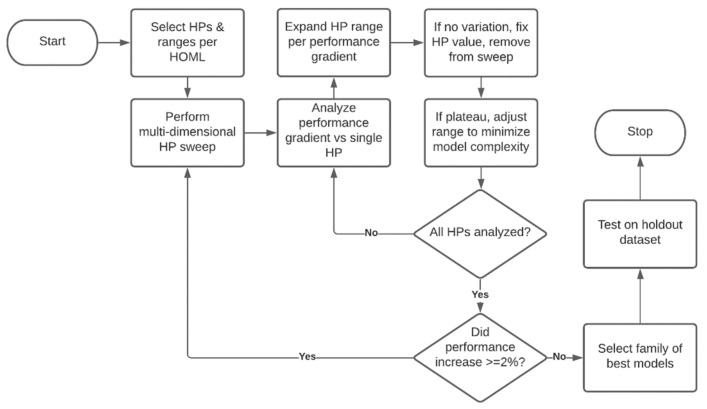
Flowchart for iterative multidimensional hyperparameter search. HP: hyperparameter; HOML: hands-on machine learning with TensorFlow [28].

An iterative multidimensional hyperparameter search was conducted that followed the process flowchart shown in Figure 3. The flowchart was executed until the model performance changed by less than 2% per iteration, and initial hyperparameters (HP) and their ranges were selected from the literature [28].

For the initial multidimensional hyperparameter search, an initial grid search sweep was performed on neurons, hidden layers, L2 regularization lambda (*λ*), batch size, epochs and learning rate, using the specifications shown in Table 1. There were 1440 combinations of hyperparameters modeled in this sweep.

**Figure 4:**
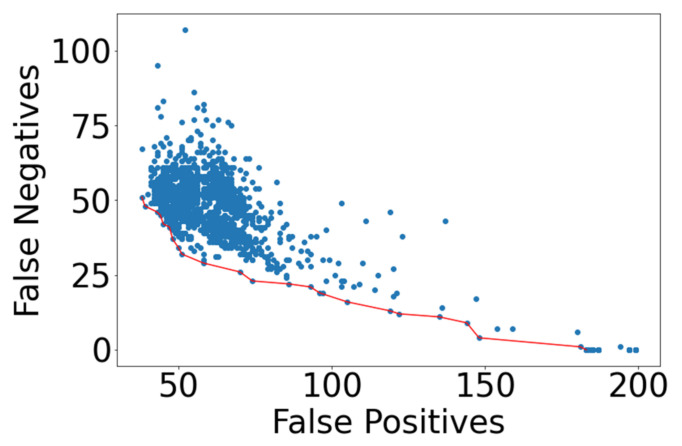
NN modeling results emphasizing lowest FN prediction results, shown by red line.

A wide range of modeling performance resulted when the counts of FN and FP were examined. In Figure 4, all model results are reported, with a red line indicating the lowest FN count for each value of FP.

**Figure 5:**
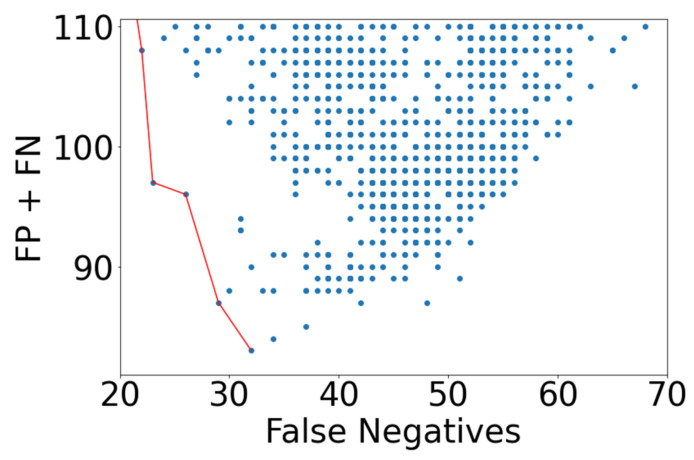
NN modeling results indicating the models with lowest FP + FN and FN counts. The red line indicates a Pareto front.

Many of the models arrived at a trivial solution, where the model over-specified FP to achieve a low FN value. This is apparent on the right side of Figure 4. More insight into modeling performance is gained when minimizing the metric “FN + FP”. This separates the trivial solutions, and is shown in Figure 5, where models that possess a FN + FP < 110 are plotted. A Pareto front is apparent which indicates the best performance for each value of false negative. 

**Figure 6:**
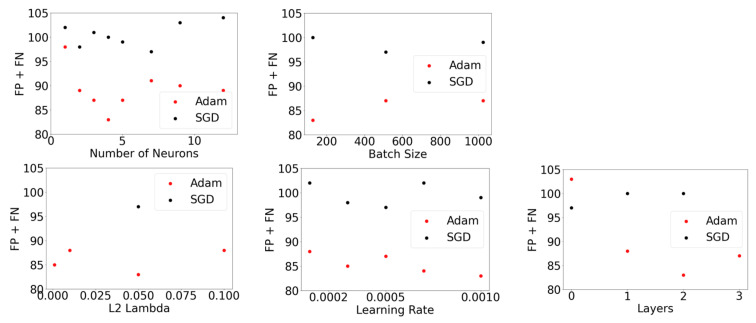
Model performance using the SGD optimizer (black markers) and Adam optimizer (red markers) in terms of FP + FN measured on the test dataset. Top panel: number of neurons and batch size; bottom panel: L2 lambda, learning rate and layers.

In order to select an optimizer, this initial hyperparameter search was repeated using the SGD algorithm where the L2 regularization sweep was replaced with SGD momentum values in the set (0, 0.3, 0.5, 0.9) An examination of each hyperparameter’s impact on modeling performance was then conducted. Figure 6 below shows a detailed analysis of the best model performance for each hyperparameter, for both the SGD and Adam optimizers, as measured by the sum of FP + FN on the test dataset.

In Figure 6, it is clear that Adam is the best optimizer for this dataset, as it has the lowest sum of false positives and false negatives. Using the Adam optimizer, the hyperparameter search ranges were modified in accordance with the Figure 3 flowchart. An example of flowchart implementation is presented by examining the Batch Size and Learning Rate subpanels of Figure 6. In these subpanels, it appears there is potential for model performance to be further improved by increasing the learning rate or decreasing the batch size. Hyperparameter search range adjustments for these two hyperparameters, and also neurons, L2 *λ* and layers were made in the second iteration of the hyperparameter search. This search was conducted using the parameters shown in Table 2. 

## 4. Discussion and Analysis

Each model was evaluated based on the FN count, accuracy, AUC score and sensitivity, which is also referred to as recall or the true positive rate. These metrics determined the model’s ability to correctly predict the risk of THC use and generalize well on unseen data.

### 4.1. Logistic Regression Model Results

For the LR model, accuracy and AUC for the train and test sets are presented in Table 3. The results show that no overfitting occurred.

**Figure 7:**
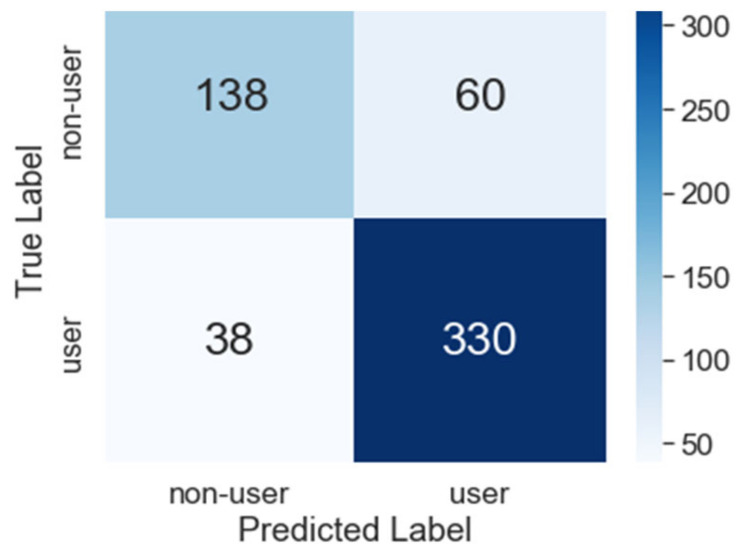
LR model confusion matrix heatmap for the test/holdout dataset.

Figure 7 shows the LR model confusion matrix that results from evaluating the model on the test dataset. The model correctly predicted 330 THC users and 138 non-users, and incorrectly predicted 38 THC users and 60 non-users. The low value of false negatives (38) aligns with the goals of this modeling effort. The receiver operating characteristic (ROC) curve for the LR model is presented in Figure 12 at the end of this section, and visually compares the true positive and false positive rates. Figure 12 also contains the results of the DT and NN models that are described below.

Comparing this LR model to those surveyed in the literature, it outperforms the model implemented by Rajapaksha et al., which achieved an overall accuracy of 0.66 and AUC score of 0.65 [5]. The present LR model is at least comparable to Spechler’s model, which achieved an AUC of 0.71 for males and 0.81 for females [6]. The study by Haug et al. reported a Nagelkerke’s R^2^ of 0.11 [7]. The closest comparison in our model is to the pseudo R^2^ result of 0.37, which is calculated as McFadden’s R^2^. Nagelkerke’s R^2^ is typically higher than McFadden’s R^2^ [29], thus we can conclude that our model outperforms this model as well.

While DT and NN models are difficult to interpret, odds ratios (OR) for the independent features are easily obtained from an LR model. The LR model did not possess the best performance of the algorithms evaluated, but it yielded valuable inferences. They are listed, with *p*-values and OR 95% confidence intervals (CI) in Table 4. 

Odds ratio values >1 indicate a positive relationship, and <1 indicate a negative relationship, and the values are constant across the range of an individual feature. For example, the 0.42 OR for Age suggests that for each unit increase in normalized age, the risk of THC use decreases by 58%. Accordingly, the 1.63 OR for *SS* suggests that for each unit increase in SS, the risk of THC use increases by 63%.

Although the OR value for Ethnicity suggests that it is the most influential variable, the wide 95% CI and skewed distribution, as seen in Figure 1, should signal using caution in this interpretation. Likewise, the variables with *p*-values > 0.05 should also be interpreted with caution.

Thus, the odds ratio offers inferences on what extent features affect the prediction of THC use. In this study, they imply that younger, less educated individuals who exhibit sensation-seeking behavior and are open to experience tend to be at higher risk for THC use. Alternatively, those who are older, more educated, agreeable, conscientious and extroverted tend to be at lower risk for THC use.

### 4.2. Decision Tree Model Results

**Figure 8:**
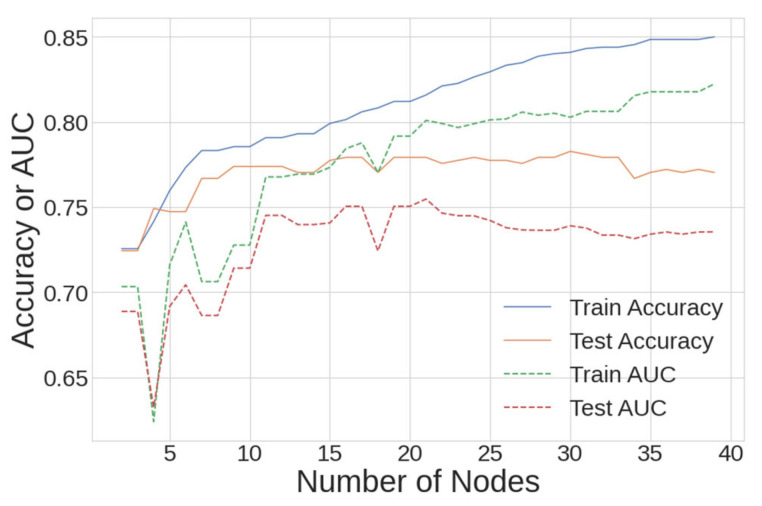
DT model accuracy and AUC vs. number of nodes for both the train and test/holdout datasets.

The resulting accuracy and AUC for the train and test datasets is presented in Figure 8 from a sweep of 2–40 leaf nodes. The criteria for the best DT model were to maximize all metrics, with more weight given to the performance on the test dataset. It was determined that 21 nodes provided the best performance with an acceptable level of overfitting, and this is where AUC_test_ had its maximum value.

The accuracy and AUC score for the 21-node DT model are presented in Table 5 for the train and test datasets, showing that a small amount of overfitting occurred. The first 4 variables that the 21-node model split on were sensation seeking, age, extroversion and conscientiousness. When compared with the four most influential variables for the LR model, based on their odds ratio and *p* < 0.05, sensation seeking and extroversion were present in both models.

The ROC curve for the DT model shows a sensitivity of 75% at a specificity of 80%, and is presented (with LR and NN curves) in Figure 12 at the end of this section. Only one study in the literature search utilized a DT model—the original study on the dataset used in this study; Fehrman et al. reported a sensitivity of 79% at a specificity of 80% [8]. Despite slightly degraded performance, the DT model in the present work is validated by reproducing prior work.

### 4.3. Neural Network Model Results

**Figure 9:**
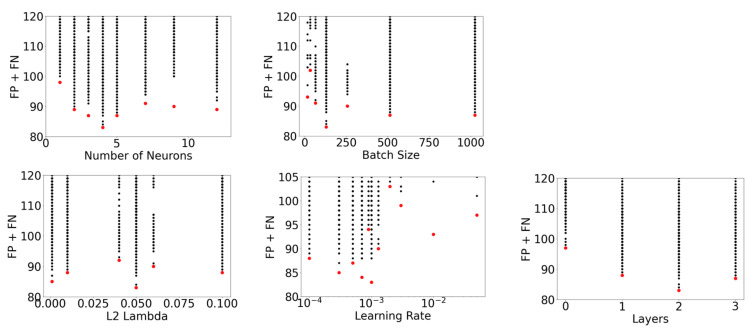
Model performance from 1D slices of the multidimensional hyperparameter search, as measured on the test dataset. Best models are indicated by a red marker, and all other models are indicated by a black marker. Top panel: number of neurons and batch size; bottom panel: L2 lambda, learning rate and layers.

Figure 9 shows the combined performance results for both iterations of the hyperparameter search, and the best performing model for each hyperparameter value is shown with a red marker. This shows the influence of five hyperparameters on model performance, as measured by minimizing the sum of false positives and false negatives on the test dataset. The epsilon hyperparameter is not shown as it did not significantly influence performance. While 4628 NN models were created, not all are visible as many had duplicate performance.

The next step in model evaluation was to select a best family of NN models and compare them to the holdout dataset. This was an important step to ensure that the model can generalize well on unseen data. The models were ranked by their accuracy metric on the entire dataset, and then the model performance was evaluated for false negatives, which is a priority for this study. Another criterion for model selection was the overfitting percentage; the f1 metric for the entire dataset was divided by the f1 metric of the test or validation datasets.

Using these criteria, a family of eight best models was selected, and their performance on the entire dataset, test dataset and holdout dataset are presented in Table 6. A hyperparameter shown in Table 6 that is not previously defined is class weight, which is the weighting factor applied to the minority class (non-user). While this can assist model performance for unbalanced datasets, the value of 1 for the best model indicates that varying this parameter did not influence optimal performance.

Models were excluded if their f1 metric on either the test or holdout set was more than 10% lower than the f1 metric on the entire dataset. This ensures that the model can generalize well on unseen data. The best model is denoted by bold text in Table 6, and while it does not have the highest f1_all_, it performs well and meets all criteria. Table 7 shows the hyperparameters of the selected model.

**Figure 10:**
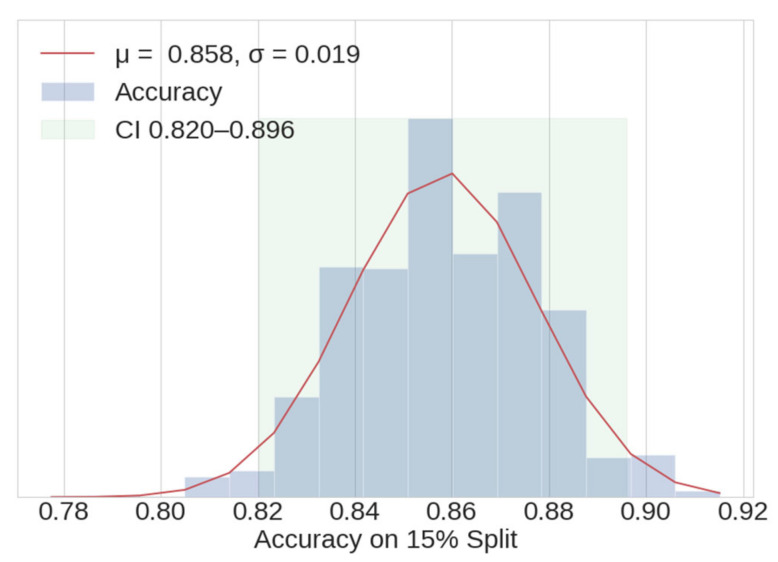
Histogram of accuracy for 1500 random splits of a 15% portion of the entire dataset.

The validation and holdout datasets each consist of a random 15% portion of the original dataset, as determined by a specified random seed. A sensitivity analysis was performed by recording the accuracy metric for a 15% split that results from 1500 different random seed values. The resulting quasi-normal histogram is shown in Figure 10, showing an accuracy mean value of 0.858 with a 95% CI of ±0.04. This gives confidence there is a relatively even distribution of outliers in the dataset; if that were not the case, the CI would be larger. As expected, the mean value of the 15% split histogram matches the accuracy of the entire dataset.

**Figure 11:**
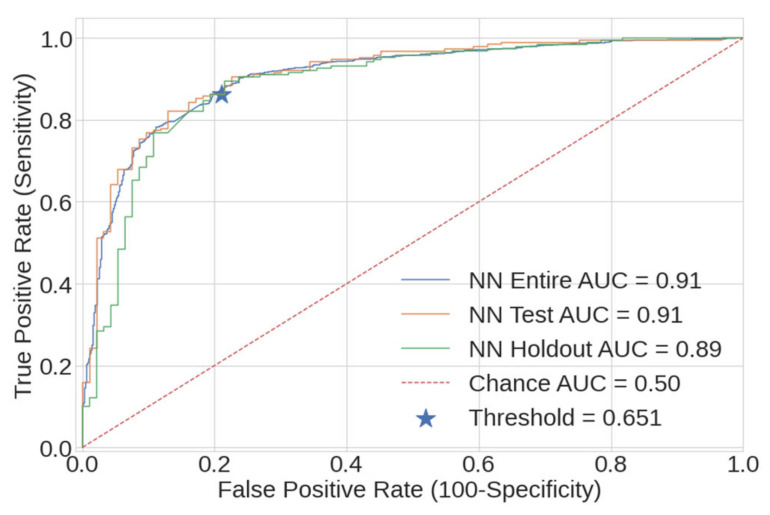
NN ROC curves, as measured on the entire dataset (blue), test dataset (orange) and holdout dataset (green). The selected specificity and sensitivity resulting from a threshold of 0.651 are denoted by a blue star.

Using the selected NN model, ROC curves were generated from the entire dataset, test dataset and holdout data, and they are presented in Figure 11. The selected specificity and sensitivity of (0.79/0.86) were selected by matching the 0.79 specificity of the prior work [8]. This pair is denoted by the blue star, and occurs at a classification threshold of 0.651.

Further metrics for the best NN model are presented in Table 8, as measured on the entire, test and holdout dataset.

### 4.4. Final Model Comparisons

Accuracy, AUC and sensitivity for the LR, 21-node DT, and NN models are summarized in Table 9. Additionally, those metrics for a hypothetical chance model (random output) and a no information rate (NIR, always predict majority class) model are presented for comparison. When compared to the LR and DT models, the NN model possessed the best AUC and sensitivity, while the LR model had the best accuracy.

**Figure 12:**
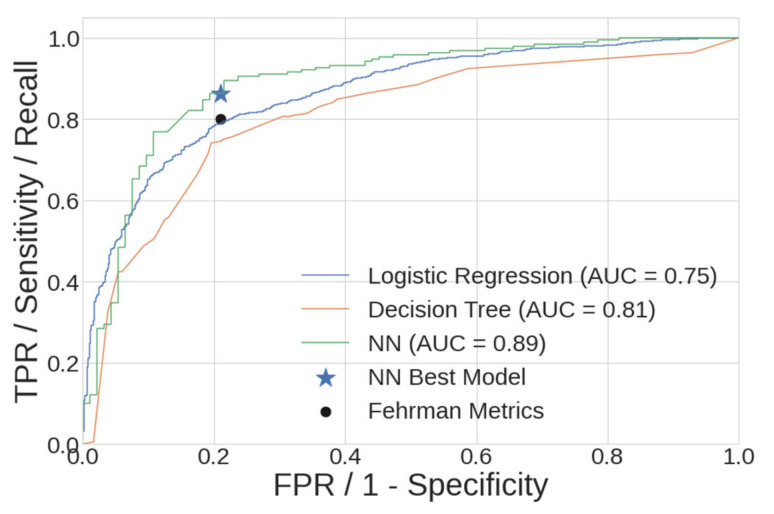
Comparison of ROC curves for the DT, LR and NN models, as measured on the holdout datasets. The sensitivity and specificity of prior work is designated by a round marker [8], and the present work is designated by a star. TPR: true positive rate; FPR: false positive rate.

The ROC curves of the three model families comprise the final comparison, as measured on the holdout datasets. This comparison is shown in Figure 12, along with the specificity/sensitivity pairs from the best NN model and those from Fehrman [8]. The increased AUC of the NN model gives the practitioner more flexibility in choosing the combination of specificity and sensitivity that are the best for their application.

As noted earlier, one goal of this study is to determine whether an NN model can produce superior results to an LR or DT model in predicting the risk of THC use. The metrics presented in this work confirmed our NN model’s ability to meet that goal. These results also validate the iterative multidimensional hyperparameter search method developed in this work. 

## 5. Conclusions

The LR and DT models developed in this work matched the performance of prior work to predict marijuana use from personality trait scores and demographics, and the NN achieved a higher level of performance. The LR model was significant (*p* < 0.05) and yielded valuable inferences by showing the extent that each feature affects the class prediction with its calculated odds ratios. The LR model showed that younger, less educated, and sensation-seeking individuals tend to be at higher risk for THC use. Older, more educated, agreeable, conscientious and extroverted individuals have a lower risk for THC use.

A method to perform an iterative method of multidimensional hyperparameter search was presented, which allowed the NN model to exceed the LR and 21-node DT performance, while avoiding a high level of overfitting. Eight optimal models from a cohort of 4600 models were evaluated on a holdout dataset, and the best NN model possessed 0.86 accuracy and 0.91 AUC on the entire dataset. The sensitivity of the present model (0.87) exceeded that of the prior work (0.80) when measured at the same value of specificity (0.79). Finally, an accuracy sensitivity analysis on a holdout-equivalent dataset yielded a 95% CI of 0.86 ± 0.04, showing a relatively even distribution of outliers in the dataset.

Given this understanding of how various personality traits may affect the risk of individuals using THC, the Air Force and the Army could possibly achieve lower numbers of THC-positive drug tests with an increased ability to focus prevention efforts. The literature shows that THC use may increase the risk for more serious opioid use disorder, and that fatalities from opioid overdoses have significantly increased in the past four decades. As a result, mitigation measures enabled by accurate THC use prediction may save service member’s lives in addition to preserving their career potential.

## Figures and Tables

**Table 1:** Neural network model initial hyperparameter search specifications.

Hyperparameter	Values Tested
Neurons	1, 2, 3, 4, 5, 7, 9, 12
Hidden layers	0, 1, 2, 3
L2 regularization λ	0.001, 0.01, 0.05, 0.1
Batch size	128, 512, 1024
Epochs	1500 with checkpointing
Learning rate	0.0001, 0.0003, 0.0005, 0.0007, 0.001

**Table 2:** Neural network model final hyperparameter search specifications.

Hyperparameter	Values Tested
Neurons	4, 5
Hidden layers	2
L2 regularization λ	0.04, 0.05, 0.06
Batch size	32, 64, 128
Adam epsilon	1 × 10^−3^, 1 × 10^−4^, 1 × 10^−5^, 1 × 10^−6^
Epochs	2500 with checkpointing
Learning rate	0.0003, 0.0007, 0.0009, 0.0013

**Table 3:** Logistic regression model performance metrics on the train and test datasets.

	Accuracy	AUC
Train set	0.79	0.74
Test/holdout set	0.81	0.77
Entire set	0.79	0.75

**Table 4:** LR model odds ratios (OR), *p*-values and 95% confidence intervals (CI). Features are listed in order of importance as determined by the OR value. *p*-values of variables with questionable significance are in red.

Feature	OR	*p*-Value	OR 95% CI
Ethnicity	6.94	0.001	2.13–22.59
Oscore	1.66	<0.001	1.37–2.01
SS	1.63	<0.001	1.30–2.05
Impulsive	1.10	0.369	0.89–1.36
Nscore	0.90	0.278	0.74–1.09
Ascore	0.86	0.067	0.72–1.01
Escore	0.81	0.048	0.66–1.00
Education	0.79	0.006	0.67–0.94
Cscore	0.79	0.014	0.66–0.95
Gender	0.73	0.082	0.52–1.04
Age	0.42	<0.001	0.35–0.52
Country	0.28	<0.001	0.21–0.38

**Table 5:** The 21-node DT model performance metrics for the train and test/holdout datasets.

	Accuracy	AUC
Train set	0.82	0.80
Test/holdout set	0.78	0.75
Entire set	0.80	0.79

**Table 6:** Hyperparameters and f1 metrics for the best family of NN models. Metrics are presented for the entire dataset (f1_all_), and the percent overfitting on the test and holdout datasets, based on the f1 metric. Bold text indicates the best model.

Neurons	Layers	L2	Learn Rate	Class Weight	f1_all_	Overfit Test	Overfit Val
10	3	1 × 10^−3^	0.01	1	0.87	17.1%	16.5%
10	2	1 × 10^−3^	1 × 10^−3^	1	0.84	10.6%	11.1%
**5**	**2**	**1 × 10^−3^**	**1 × 10^−3^**	**1**	**0.84**	**7.3%**	**8.4%**
5	2	1 × 50^−3^	0.01	1.5	0.82	5.9%	8.9%
4	2	1 × 50^−3^	0.01	1.25	0.82	4.9%	8.1%
5	2	1 × 50^−3^	0.01	1.25	0.82	2.0%	6.6%
5	2	0.01	0.01	1.75	0.82	7.1%	8.3%
5	2	0.02	1 × 10^−3^	2	0.80	4.9%	4.7%

**Table 7:** Neural network model final hyperparameters. AF: activation function.

Parameter	Specification
Input layer/AF	23 neurons/ReLu
Hidden layer/AF	2 layers, 5 neurons/ReLu
Output layer/AF	1 neuron/sigmoid
L2 regularization *λ*	0.001
Batch size	512
Epochs	1500 with checkpointing
Optimizer algorithm	Adam
Adam epsilon	0.0001
Learning rate	0.001
Loss function	Binary cross-entropy

**Table 8:** Metrics for the best NN model.

Dataset	AUC	Accuracy	Specificity	Sensitivity
All	0.91	0.86	0.79	0.87
Test	0.91	0.79	0.79	0.86
Holdout	0.89	0.78	0.79	0.86

**Table 9:** Model metric comparisons, as measured on a 30% holdout dataset (DT and LR) or a 15% holdout dataset. NIR: no information rate, or the metrics that result from always predicting the majority class of “THC user.”

Metric	LR	DT	NN	Chance	NIR
Accuracy	0.79	0.76	0.78	0.45	0.72
AUC	0.75	0.81	0.89	0.50	---
Sensitivity	0.80	0.76	0.86	0.68	1.00

## Data Availability

The dataset is publicly available [24].

## Authors’ Note

The views and opinions of authors expressed herein do not necessarily state or reflect those of the U.S. Government or any agency thereof. Reference to specific commercial products does not constitute or imply its endorsement, recommendation, or favoring by the U.S. Government. The authors declare this is a work of the U.S. Government and is not subject to copyright protections in the United States. This article was cleared with case numbers 2021-0292, MSC/PA-2021-0167 and 88ABW-2021-0421.
